# Too much of a good thing? Russia-EU international trade relations at times of war

**DOI:** 10.1007/s40812-022-00232-2

**Published:** 2022-09-25

**Authors:** Lucia Tajoli

**Affiliations:** Department of Management, Economics and Industrial Engineering, Politecnico di Milano, Milan, Italy

**Keywords:** Economic integration, Specialization, Centrality, Global value chains, Russia-Ukraine war, F14, F15

## Abstract

In this paper, we investigate the evolution of Russia’s position in the world trade system, especially in relation to the European Union (EU). Data show that after entering into the WTO, Russia did not use this accession to develop and diversify trade flows (like China did, for example) but it augmented its specialization in fuels and raw materials, increasing its dependency on the rest of the world, and especially on European demand. Russia did not exploit its trade potential and its favorable geographic position to foster its economic development and to improve the welfare of its population. At the same time, the integration within the European Single Market and with the rest of the world both for older and new EU member formerly linked to the Soviet Union, has helped the EU to maintain high standards of living, and a relative stability, even if increasing its dependency on Russian fuels. We argue that it is also because of these differences and the related economic problems that tensions between Russia and the EU grew over time.

## Introduction

Since the fall of the former Soviet Union, Russia’s economy, growth and international trade followed an uneven path, especially compared to other so-called “transition economies”. The transformation of a country from a centralized economic system into a market economy is far from straightforward and easy, as a large number of studies have shown.^1^ In the case of Russia, a very large country lacking more than others market foundations and capitalist institutions, this has proven even more complex (Ericson, 1991, 1999). The reliance on its huge natural resources brought about the under-development of a number of manufacturing sectors, some of them formerly quite advanced.

As effectively summarized by Alexeev and Weber (2013):

*After a steep decline during most of the 1990s, Russia’s economy was growing at almost 7 percent beginning in 1999 ….. Although the impressive economic growth since 1999 has raised the standards of living and put scores of Russians on the Forbes billionaires list, it has not solved a number of deep economic and social problems …. The country continues to suffer from low labor productivity, distorted and undiversified structure of the economy, with its heavy reliance on natural resource extraction, low life expectancy, high income inequality, and weak institutions, including pervasive corruption and poor property rights protection.*

(from the Introduction to The Oxford Handbook of the Russian Economy).

In a sort of “Dutch disease” kind of effect^2^ (Corden & Neary, 1982; Sachs & Warner, 1995, Behzadan et al., 2017), the curse of natural resources and especially fossil fuels abundance, together with the lack of many important institutions necessary for the proper functioning of a market economy have hampered economic development and growth in Russia. The reliance on fuels is spread throughout the Russian economy, and it is not only affecting manufacturing production and trade: current estimates indicate that revenues from oil exports make up 40% of Russia’s federal budget (Gordon, 2022).

The fragile evolution of the Russian economy and trade is associated with the position taken by the Russian government in international affairs. Previous studies have highlighted the existence of a relationship between economic interdependence and the peaceful or belligerent attitude of countries, starting from Keynes (1919). More recently, Martin et al. (2008) and Jackson and Nei (2015) show empirically that this relationship is more multifaceted than expected. Analyzing international relations over two centuries, Copeland (2015) demonstrates the crucial role of expectations on the trade environment for countries’ relations. According to this analysis, when leaders have positive expectations of the future trade environment, they want to remain at peace in order to secure the economic benefits that enhance long-term power. When, however, these expectations turn negative, leaders are likely to fear a loss of access to raw materials and markets, giving them more incentive to initiate crises to protect their commercial interests. We argue that the weak position of Russia in international trade and the associated lack of an “economic superpower” status are among the reasons that explain Russia confrontational attitude toward its neighbors.

The European Union (EU) relation with Russia also followed an irregular path, alternating moments of tighter economic integration with political crises. Often, short-term economic convenience, for example in terms of quick availability of gas and oil, has shadowed the risk inherent in the Russian market for European firms, and in the lack of diversification of suppliers.

Consequently, what we observe now is a very unstable situation, with a reciprocal dependence. In this paper, we aim to show how on the one hand, the reliance on a large market for fuels has hindered the stable economic development of Russia and the expansion of its trade position, creating an uneven relationship with the EU, and potentially weakening Russia’s interest in a stable economic environment. On the other hand, this situation determined a (much weaker) dependence on Russia’s fuel in some EU sectors. This uneven situation is unlikely to foster economic development and stable trade links, while it is expected to bring about tension or possibly conflicts, as we have observed in the past months.

## Russia’s position in international trade

### Overall trend and specialization

**Fig. 1:**
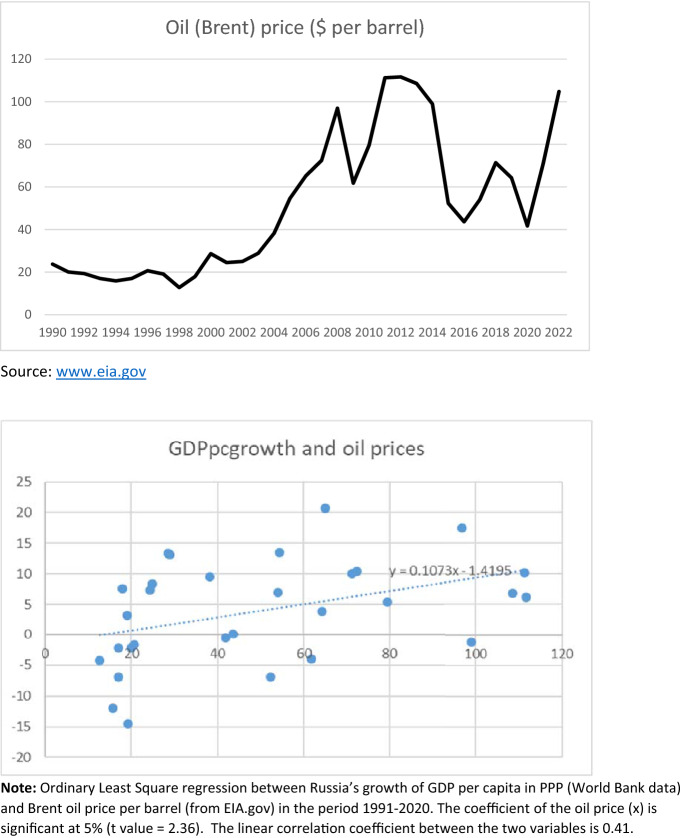
Correlation between Russia’s GDP growth and oil prices

Russia has been the largest so-called “transition economy”^3^ and in this process it followed an uneven path. After a decline in gross domestic product (GDP) per capita lasting from 1991, when the Union of the Socialist Soviet Republics (USSR) was dismantled, until 1998, the economic growth of Russia turned again positive, but with many fluctuations (see Table 1). In the past decades, GDP per capita yearly growth rates appears highly correlated to its fuels exports and their prices (see for example Beck et al., 2007, Kuboniwa, 2012, and Fig. 1).^4^ This is not surprising, given the relevance of Russia’s trade specialization on oil and gas. The weight of fuels on the total value of Russian exports increased from about 45% in the late 1990s to a peak of 70% in 2013 to eventually decline to 52% before the pandemic crisis in 2019. Only a few countries in the world rely on fuel exports more than Russia, and they are the “usual suspects”, mostly in the Arabic peninsula or Central Asia.

**Table 1:** GDP disparities: GDP per capita in Russia (in bold) and in selected countries

Year	1992	2000	2005	2010	2015	2016	2017	2018	2019	2020
Country										
Russia	**6862.4**	**6825.4**	**11,822.3**	**20,490.1**	**24,085.3**	**24,128.1**	**25,926.4**	**28,821.2**	**29,967.1**	**29,812.2**
European Union	15,958.0	22,073.7	26,815.1	32,865.7	38,222.8	40,580.3	42,676.6	44,640.0	46,069.8	44,763.2
Bulgaria	6916.2	6422.0	10,291.4	14,956.5	18,391.9	20,074.4	21,469.9	22,990.1	24,523.8	24,613.8
Czech Republic	11,841.9	16,235.5	22,095.4	27,882.0	33,899.3	36,097.7	38,824.9	41,135.5	42,847.0	41,604.0
Estonia		9421.3	16,634.9	21,619.5	29,175.9	31,312.8	33,821.9	36,249.4	37,850.1	37,600.6
Hungary	8252.9	11,853.2	17,112.2	21,751.8	26,806.6	27,947.6	29,501.1	31,913.1	33,514.9	33,077.2
Lithuania		8445.9	14,510.5	20,096.7	28,834.4	30,925.2	33,761.9	36,376.2	38,540.8	38,883.1
Latvia		8039.5	13,913.8	17,706.9	24,972.8	26,721.7	28,673.6	30,877.0	31,883.3	31,464.5
Poland	6189.8	10,672.1	13,896.8	21,072.5	26,862.1	28,322.1	30,064.5	31,953.0	33,797.8	34,240.2
Romania	4512.6	5848.1	9602.1	16,976.3	21,605.8	24,271.5	27,141.9	29,309.2	31,901.4	32,116.5
Slovak Republic	7172.3	11,376.6	16,639.4	25,302.2	29,964.9	29,645.7	30,061.6	31,214.6	31,966.6	31,356.5
Slovenia		18,001.2	23,852.6	27,826.9	31,628.2	33,936.0	36,507.6	38,961.5	40,670.9	39,768.6
Ukraine	6592.6	4265.1	7210.6	8559.0	10,164.3	11,148.2	11,860.6	12,633.1	13,346.5	13,054.8
China	1262.6	2920.6	5053.9	9253.8	12,897.5	13,483.4	14,243.5	15,497.4	16,653.3	17,210.8

Gross Domestic Product (GDP) per capita values in Purchasing Power Parity (PPP), current international $

Source: World Bank, World Development Indicators database

Benedictow et al. (2013) develop a macroeconometric model showing that the Russian economy is vulnerable to large fluctuations in the oil price, even if there is evidence of significant economic growth capabilities in the absence of oil price growth. They find that higher oil price leads to higher economic growth and savings in the sovereign wealth fund, but also to breaches in the Russian economy, as the traditional export industries suffer from real appreciation, in line with the Dutch disease hypothesis.

**Fig. 2:**
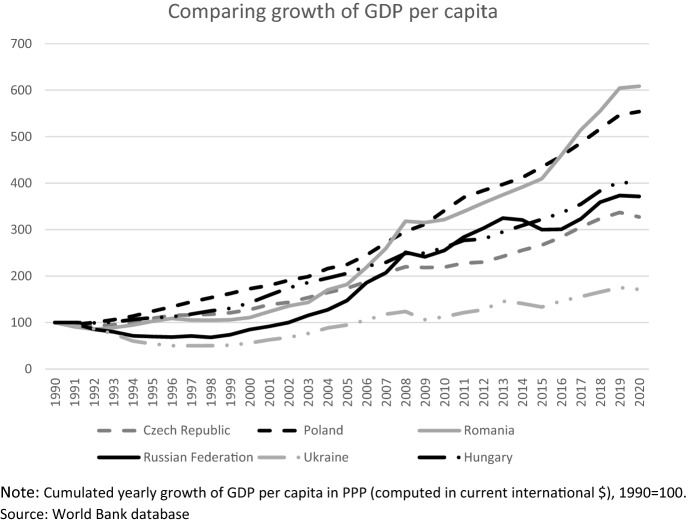
GDP per capita growth trend in Russia and in selected Central and Eastern European Countries (Source: elaborations on World Bank data)

Because of this unstable growth path, Russia did not display a significant catching up in terms of per capita GDP with respect to the European Union (Table 1). As shown in Fig. 2, the trend of Russia’s growth is outperformed by many other former “transition economies” in Central and Eastern Europe (Svejnar, 2002). The group of CEECs,^5^ formerly closely linked to the USSR, started to develop close economic ties with Western Europe in the early 1990s, and then became members of the EU in 2004 and 2007. In the effort to meet the strict conditions imposed to join this treaty, they deeply transformed their economies and rapidly increased trade and foreign investments with the other member countries (Hoekman & Djankov, 1997; De Benedictis & Tajoli, 2008). According to many studies, trade integration played a crucial role in the economic transformation and in the transition process (see for example Atrupane et al., 1999; Ofer & Drebentsov, 1999; McMillan and Woodruff, 2002; Gorodnichenko et al., 2010), and overall this produced a much stronger and stable growth path and a faster catching up.

Russia was not initially involved in this integration. Its size, its economic and political history and its national policy did not make it a candidate to join a trade agreement with the EU. But the importance of trade integration both for economic and political reasons, pushed Russia to sign trade agreements with countries formerly belonging to the USSR, and to apply to join the World Trade Organization (WTO) in the mid-1990s (Isachenko, 2013), also in an effort to attract foreign investors and to develop a modern manufacturing sector. The goal of WTO membership was reached only in 2012, also because of many political tensions with other members of the WTO that vetoed its entrance for a decade.

Even entering into the WTO and the consequent easier access to foreign markets did not significantly change Russia’s trade position and specialization. On the one hand, Russia experienced a negative demographic trend,^6^ that did not allow to rely on an abundant labor force to develop comparative advantages in labor-intensive sectors, similarly to what China did in its early trade development stages. On the other hand, the obsolete domestic capital and manufacturing structure (Ericson, 1999) and the difficulties in attracting foreign capitals because of the high level of uncertainty and corruption, and weakness of many institutions (Brock, 1998; Buiter, 2000) did not allow to build significant comparative advantages in advanced capital-intensive sectors either.

**Fig. 3:**
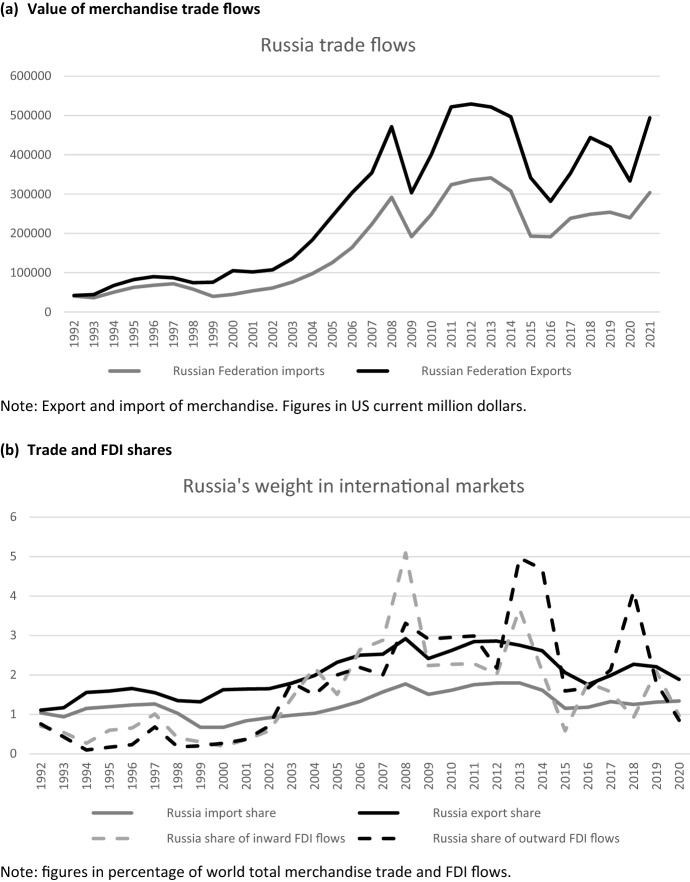
Trends in Russia's trade (values in panel a, shares in panel b) and foreign direct investments (shares in panel b) Source: UNCTADstat

Russia’s commitments were meant to progressively and significantly lower the applied tariffs of the Russian Federation (Shepotylo & Tarr, 2013), and provide better access to international markets for Russian products. Russia’s accession to the WTO raised the expectations that trade with Russia would benefit from sustained liberalization. Instead, Russia has progressively put in place numerous measures favoring domestic products and services over foreign ones, and incentivizing localization of production in Russia by foreign companies (Connolly & Hanson, 2016; Erokhin, 2017). This import substitution policy has been continually expanded, and therefore it is not surprising that the effects of WTO accession are hardly visible in trade data. Figure 3 shows the increasing trend of Russia’s trade in the first years of the current century, but the removal of trade barriers after 2012 had a very small impact, reversed by the effects of sanctions on Russia afterward Crimea’s invasion in 2014. Looking at Table 2, we see that the Russian share of world export increased after the 1990, but it certainly did not improve after joining the WTO. Once more, the observed changes in Russia’s export share are very much linked to the fluctuation in fuels prices.

**Table 2:** Countries’ shares in world merchandise exports (top 15 countries in 2021, Russia's figures in bold)

Year	1992	2000	2010	2018	2019	2020	2021
Economy							
China	2.24	3.86	10.31	12.72	13.15	14.68	15.10
USA	11.83	12.12	8.36	8.51	8.65	8.08	7.87
Germany	11.36	8.53	8.23	7.98	7.84	7.84	7.32
Netherlands	3.71	3.60	3.75	3.72	3.73	3.82	3.75
Japan	8.98	7.43	5.03	3.78	3.71	3.63	3.39
China, Hong Kong SAR	3.16	3.14	2.62	2.91	2.81	3.11	3.01
Korea	2.02	2.67	3.05	3.09	2.85	2.90	2.89
Italy	4.70	3.73	2.92	2.81	2.83	2.83	2.74
France	6.25	5.06	3.42	2.98	3.00	2.77	2.63
Belgium	3.25	2.91	2.66	2.40	2.35	2.39	2.44
Canada	3.55	4.29	2.53	2.31	2.35	2.22	2.26
Mexico	1.22	2.58	1.95	2.31	2.42	2.36	2.22
** Russia**	**1.11**	**1.63**	**2.62**	**2.27**	**2.21**	**1.89**	**2.22**
United Kingdom	5.05	4.41	2.72	2.49	2.42	2.26	2.10
Singapore	1.68	2.14	2.30	2.11	2.06	2.05	2.05

Source: UNCTADstat

**Fig. 4:**
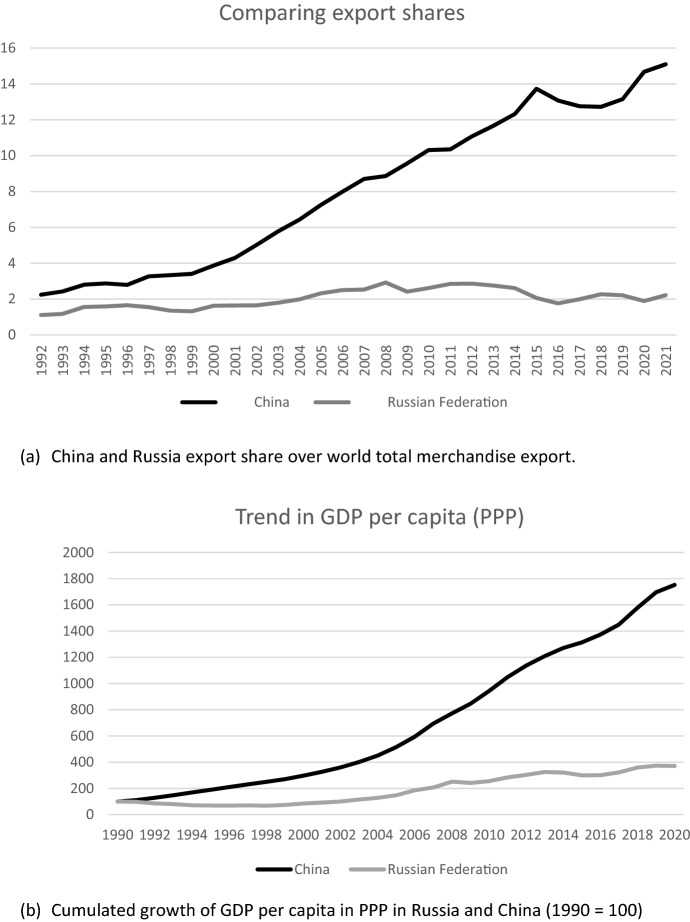
Russia and China trade shares (panel a)  and GDP per capita growth (panel b) Source: UNCTADstat and elaboration on World Bank data

The figures reveal an evolution very different from the one of China (Buck et al., 2000), who fully exploited its entrance in the WTO in 2001, rapidly becoming the largest world exporter, and increasing the average GDP per capita of its population (see Fig. 4).^7^ Nothing comparable happened to the Russian economy. By the time the WTO membership became effective, the economic integration with the EU and with the more advanced countries was no longer a priority of the Russian government, and the attitude toward the WTO rules was quite conflictual, overcome by other policy priorities, opening a number of disputes with other countries, especially the EU and the USA (Neuwirth & Svetlicinii, 2016). There are no signs that WTO membership has significantly changed Russia’s attitude toward multilateral trade rules (see Aaronson & Abouharb, 2014).

Some studies examined the impact of Russia’s entrance into the WTO on its trade. Cristea and Miromanova (2022), using customs data on the monthly import and export transactions of Russian firms over the period 2011–2015, investigate the short-run responses of firm-level trade along the intensive and extensive margins following Russia’s WTO accession in 2012. They find that over this period, there was an increase in the number of foreign countries that Russian firms export to or import from, and a significant increase in the number of exported products, but the evidence on the effects of the WTO accession on the intensive margin of firm-level trade is mixed. It is also important to remember that Russia’s trade was further hampered by the (mild) sanctions that hit the country in 2014 after the invasion of Crimea. In response to these sanctions, the Russian government imposed a retaliatory food embargo, which might have been intended as a protectionist policy to help the vulnerable domestic agricultural sector directly impacted by the country’s WTO accession.

If we consider the revealed comparative advantages (RCAs) of Russia in international trade, measured using the Balassa index (Balassa, 1965), we see that data confirm very little change before and after the entrance into the WTO. Table 3 displays the RCA indices of Russia (computed as the Russian export share in a given product over the overall Russian share in world exports) for the ten products in which the country reveals the strongest comparative advantage. With the exception of wheat, these are quite stable since the 1990s. The table also reports the few products in which a stable revealed comparative advantage appears after 2012, that is after joining the WTO. Overall, Russia’s comparative advantages are quite concentrated in fuels and raw materials, a few agricultural products and some basic industrial products. Russia does not show a RCA in over 200 products out of 260 and this did not change in the past decade. This evidence seems to confirm the hypothesis of a Dutch disease effect and the ineffectiveness of the formal opening in changing the specialization pattern.

**Table 3:** Russia’s industries of revealed comparative advantages

Year	1995	2000	2012	2015	2016	2017	2018	2019	2020
Product									
**[672] Ingots, of iron or steel**;	10.41	10.23	7.02	10.35	11.92	9.65	9.63	9.19	15.19
[041] Wheat (including spelt), unmilled	0.22	0.19	3.21	4.82	6.01	6.83	8.78	6.95	9.37
**[321] Coal, not agglomerated**	3.08	4.24	3.60	6.00	6.35	5.66	5.75	6.23	8.88
** [562] Fertilizers (other than group 272)**	6.29	7.66	5.27	6.59	8.75	8.02	5.86	6.17	7.97
**[248] Wood simply worked, and railway sleepers of wood**	1.38	1.76	3.31	3.94	4.53	4.46	4.30	5.00	6.59
**[333] Petroleum oils, crude**	4.37	3.96	3.68	5.38	6.01	5.24	4.93	5.22	6.57
[043] Barley, unmilled	0.73	0.96	3.97	5.59	3.54	5.11	5.60	4.88	6.56
[284] Nickel ores and concentrates;	0.03	0.03	0.07	0.12	2.66	5.17	5.04	5.81	6.05
**[334] Petroleum oils > 70% oil**	4.56	4.09	3.56	5.21	5.67	5.00	4.14	3.96	5.91
**[671] Pig iron and spiegeleisen, sponge iron**	3.53	3.87	3.93	5.21	5.59	4.71	4.71	4.41	5.91
[223] Oil seeds and oleaginous fruits	0.25	0.29	1.86	2.38	3.48	2.91	1.81	2.83	3.01
[679] Tubes, pipes and hollow profiles,	0.72	0.85	0.69	0.67	0.88	1.40	1.43	1.27	2.44
[091] Margarine and shortening	0.14	0.35	1.00	1.37	1.55	1.48	1.31	1.55	2.12
[343] Natural gas, whether or not liquefied	23.55	14.79	6.51	8.84	1.04	0.89	1.10	1.87	1.84
[246] Wood in chips and wood waste	0.51	0.54	1.27	1.21	1.40	1.44	1.40	1.84	1.70
[685] Lead	0.20	0.04	1.12	1.42	1.93	1.60	1.29	1.11	1.66

There is a revealed comparative advantage (RCA) in a given product if the RCA index is > 1. Products in bold are the ones with a revealed comparative advantage  for the entire period. The first ten products are the ones with the highest RCA in 2020 in the three-digit level of the SITC commodity classification, Revision 3, which includes about 260 products. The remaining reported products are the ones where a new stable RCA appears in the last decade

Source: elaboration on UNCTADstat data

Russia is also a relevant exporter of materials used in the production of fertilizers, and a large exporter of critical raw materials, such as palladium, vanadium, and cobalt, which are most prominently used in a number of high-tech industries like 3D printing, drones, robotics industries, batteries, and semiconductors. Thus, Russia is in the position of affecting production also in other sectors, such as electronic appliances, transportation, and most prominently the car sector, but it never developed sufficient domestic production capability in these downstream industries so to grasp potentially relevant economic benefits.

A possible effect of the improved integration of Russia within international markets during the negotiations for the WTO accession was the increased attractiveness of Russia for foreign direct investors. For a few years, from 2000 to 2008 the share of foreign direct investment flows directed to Russia increased remarkably, but this effect was temporary. After the great financial crisis and the invasion of Crimea, the perceived country risk was again very high, discouraging many investors (Domínguez-Jiménez & Poitiers, 2020). Now Russia ranks around the 20th position in terms of share of received FDIs, according to UNCTAD data.

### Russia’s role in the world trade network

A different way to assess a country position in the world trading system is to measure its centrality in the network formed by countries (the network nodes) and trade flows (the edges or links of the network). In a recent study, De Benedictis and Tajoli (2018) show how emerging countries occupying a more central position in the world trade network seem to perform better in terms of trade and growth, and regression analysis confirms a positive relationship between network centrality and growth for emerging countries.^8^ The centrality of a node in a network can be measured in many different ways, locally and globally, but a relevant class of measures in the context of trade relations are the ones using eigenvector centrality. Differently from local centrality measures, that identify how many trade links a country has, or how strong those links are in terms of the value of trade (a measure that can be shown to be equivalent to the export market share of a country), the basic concept of a node's eigenvector centrality is that this measure associates node’s centrality to the node neighbors' characteristics, directly referring to how important, central, influential or tightly clustered a country’s trade partners are.

With this global measure, the whole structure of the network is taken into account, and it is not the country's centrality in itself that matters, but the centrality of the countries it is linked to (see De Benedictis et al., 2014). In other words, to be central in trade according to this measure, a country should be connected to important traders, and it is this type of centrality that helps economic growth. In principle, given its geographic proximity to the European Union and its historical ties with the European culture, based on the definition of eigenvector centrality, we would expect a high centrality for Russia.

Table 4 reports the computed PageRank centrality measure for Russia over time.^9^ The value taken by this index depends on the overall centralization of the entire network, which in the case of the world trade network tends to decline in the past decades. The most central country since the mid-1990s is the USA, with values of the index ranging approximately between 0.14 and 0.11. Until 2009, the second most central country was Germany, with index values around 0.7–0.6, who yielded the second position to China in 2010, with values around 0.8. It is evident that in spite of its geographic position and the links with a central country like Germany, and with other EU members, Russia’s centrality is not high, moving around 0.01, and declining in the past decade. In particular, as a major oil and gas exporter, Russia has a significantly higher centrality score considering outgoing links (exports) rather than incoming links (imports). We can also observe that its centrality score over time is positively correlated with a value of 0.57 with the fuel share in Russia’s exports.

**Table 4:** Centrality of Russia in the world trade network

Period	Rank	Page Rank Index value
1996–1997	16	0.014843
1998–1999	18	0.010782
2000–2001	22	0.009684
2002–2003	19	0.009978
2004–2005	19	0.011411
2006–2007	16	0.014542
2008–2009	17	0.015048
2010–2011	17	0.015021
2012–2013	18	0.015821
2014–2015	19	0.012387
2016–2017	22	0.011237
2018–2019	21	0.011366

Source: Author’s elaboration on CEPII BACI data

Russia’s centrality can be examined not only with respect to the entire world trading system, but also with respect to Russia’s regional network. This was done in a study on the structure of regional networks by Iapadre and Tajoli (2014), considering the Commonwealth of Independent States (CIS) as the reference region for Russia in the 20 years after the dismantling of the Soviet Union. This area was characterized in 1995 by extremely high rates of intra-regional trade preferences, as the former ties between the Soviet Republics were de facto still present to a large extent. Constrained by the problems created by the transition to the market system, especially in the earlier phases of transition, CIS countries tended initially to trade almost exclusively between each other, following the patterns of their previous regimes, but the opening to extra-regional trade has been very rapid in the following decade. Russia has hastily assumed the lead of this process, playing the role of a dominant local supplier. In 2011, while Russia global centrality is quite low, even if slightly increased from the previous decade, its regional centrality is very high. Within the CIS, Russia’s trade centrality is challenged only to some extent by Ukraine and Belarus, and the overall centralization indices are very high, displaying very asymmetric positions within the region, with some countries appearing as truly peripheral. Also at the regional level, the centrality of Russia is linked essentially to its role as an exporter, while the indices as importer are much lower.

### Russia’s participation in Global Value Chains

The scarce development of Russia’s comparative advantage in the manufacturing sectors and the increasing reliance on fuels’ exports have affected also Russia’s participation in Global Value Chains (GVCs). The involvement of this country has been remarkably different from the one of other emerging and transition economies, like the CEECs, which started very early in their transition to be integrated in the European production networks, well before joining the EU (see for example Baldone et al., 2001). Participation in the European production processes has deeply affected the evolution of the CEECs’ specialization, and spurred their convergence with the rest of the EU (De Benedictis and Tajoli, 2008).

In the past decade, a set of indicators has been developed to measure countries’ participation in GVCs (see Koopman et al., 2014; Borin & Mancini, 2019; Borin et al., 2021). If we consider the so-called backward GVC participation index, i.e. the foreign value added share of gross exports, we see that many CEECs are fully involved in the manufacturing process of many European industries. Czech Republic, Hungary, Poland use large amounts of foreign value added in their exports because a large share of their exported products are obtained joining the production chains of other countries, primarily Europeans (see Table 5). Most EU members are highly involved in European GVCs, regardless of their average income levels: Germany and Italy use a considerable amount of foreign value added in their exports as well. This is not the case for Russia, whose participation in international manufacturing production is quite low, as the backward participation index shows for both types of decompositions (Panel a and b of Table 5).

**Table 5:** Countries’ participation to Global Value Chains

(a) Comparison of countries’ GVC participation (Koopman et al. (2014) decomposition)												
	Backward GVC participation (FVA sh.)				Forward GVC participation (DVAFX share)				Total GVC participation			
	1995	2000	2010	2018	1995	2000	2010	2018	1995	2000	2010	2018
Czech Republic	24.7	29.4	40	42.2	16.4	18.8	18	19.7	41	48.2	58.1	61.8
Hungary	26.8	46.5	48.3	46.3	12.3	11.5	13.7	17	39.1	58	62	63.3
Poland	16.2	23.6	29.7	31	15.9	20.7	20.7	22.6	32.1	44.3	50.5	53.7
Romania	18.9	17.4	21.3	24.4	15.2	19.9	20.6	23.3	34.1	37.3	41.9	47.7
Germany	14.0	18.8	22.7	22.9	19.4	21.1	21.4	23.4	33.5	40	44.1	46.3
Italy	16.4	18.9	24.1	23.2	13.9	16.4	17.6	19.9	30.3	35.4	41.7	43
Russia	9.2	9.8	8.5	8.6	24	29.5	36.3	37.1	33.2	39.3	44.8	45.6
China	15.8	17.5	19.2	17.2	12.6	15.1	17.5	19.3	28.4	32.6	36.7	36.6

(b) Russia GVCs participation (Borin et al., 2021 decomposition)						
Indicators (Percent of trade)	Years	2016	2017	2018	2019	2020
Russia	Pure forward GVC participation (GVCPF)	36.31	37.85	43.09	32.21	30.98
Russia	Pure backward GVC participation (GVCPB)	4.92	4.97	5.51	7.05	7.11
Russia	Two-sided GVC participation (GVCBF)	2.98	3.20	4.23	3.35	3.10

Source: TiVA OECD database (a)

Source: World Bank WITS database (b)

**Fig. 5:**
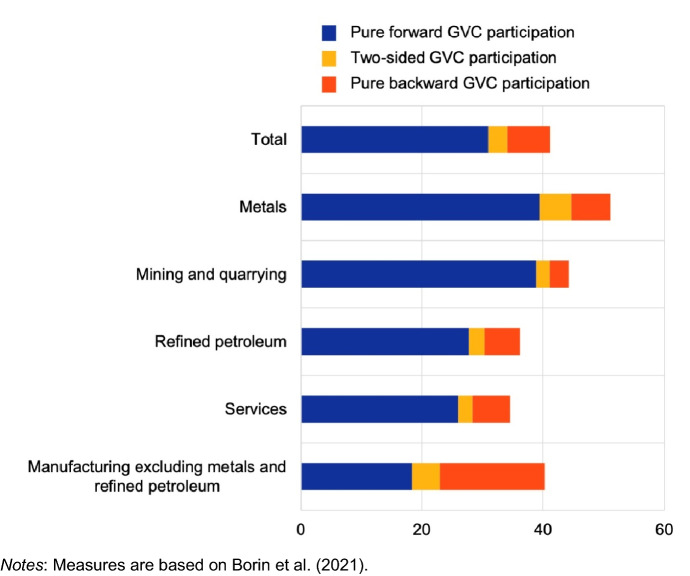
Russia’s participation in global value chains across sectors by different modes (percentage share of total exports, 2020). Source: Borin et. al (2022) using World Bank WITS, ADB MRIO tables. Notes: Measures are based on Borin et al. (2021)

The forward GVC participation index corresponds to the ratio between the domestic value added sent indirectly to third economies and the economy's total gross exports. It captures the domestic value added contained in inputs sent abroad for further processing and exported to third economies through supply chains. We see that in advanced economies, well integrated in the world production system, like Germany and Italy, the value of the forward participation index is close to the one of backward participation. For the CEECs, holding a different position in GVCs, the forward participation index is lower, but not negligible. Instead, Russia shows a high value of forward GVC participation, as more than 30% of its exports consist of inputs used by its trade partners as intermediate inputs, compared to a global average of about 18%. This is explained by Russia’s specialization in oil, gas and metal industries (Fig. 5), used as inputs in other countries’ productions and intrinsically more forward integrated, being positioned upstream in the production process. Such a strong asymmetry in participation to GVC is typical of fuel exporting economies. Even China shows a much more balanced forward and backward involvement in GVCs.

**Fig. 6:**
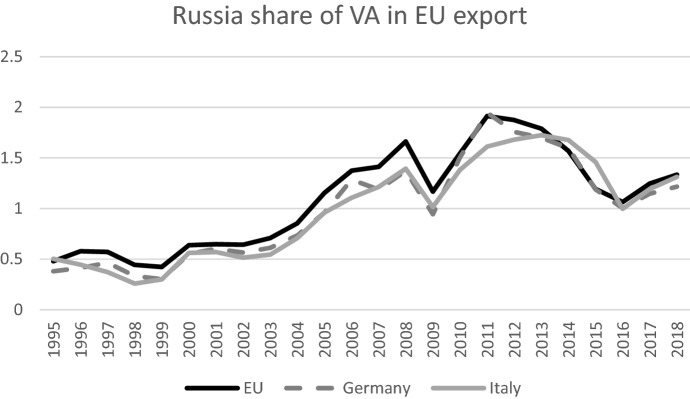
Russian value added share in EU gross exports. Source: OECD TiVA database

Considering specifically the role of Russia in EU GVCs, we see that the Russian value added contribution is small, and it amounts to approximately 1.3% of the value of EU exports (see Fig. 6). A similar share is observed also for the two main EU exporters, Germany and Italy, with a Russian value added share in their exports of 1.3 and 1.2 respectively. This share has been increasing between 1995 and 2005 and it then fluctuated between 1 and 2%, following closely the fluctuations of the oil price.

This limited and one-sided involvement of Russia in GVCs can be related to the above-mentioned weak comparative advantages in many manufacturing sectors. Another important factor that can explain the low value of Russia’s backward GVC indicators are the country’s feeble institutions and problems with rule of law and contract enforcement (Buiter, 2000). As extensively discussed in the literature on GVCs, especially in case of advanced and complex productions, the reliability of intermediate suppliers and contracts implementation are crucial for a firm to decide where to delocalize some production phases (Antràs & Chor, 2021; Antràs & Helpman, 2004). Russia’s low reliability in this respect made many firms prefer other emerging countries as suppliers and production locations.

Given the upstream position of Russia in GVCs, disruptions to Russia’s exports might well propagate downstream through supply chain networks, having an impact also via the indirect trade (Winkler & Wuester, 2022; Winkler et al., 2022). But this type of participation in GVCs based on commodities implies a very limited control on downstream production and prices, and on the overall value added of the entire production process. Furthermore, the lack of active participation in manufacturing GVCs implies a lower exposition to foreign technology and know- how, with fewer knowledge spillovers (Tajoli & Felice, 2018).

### Russia and EU trade relations

**Fig. 7:**
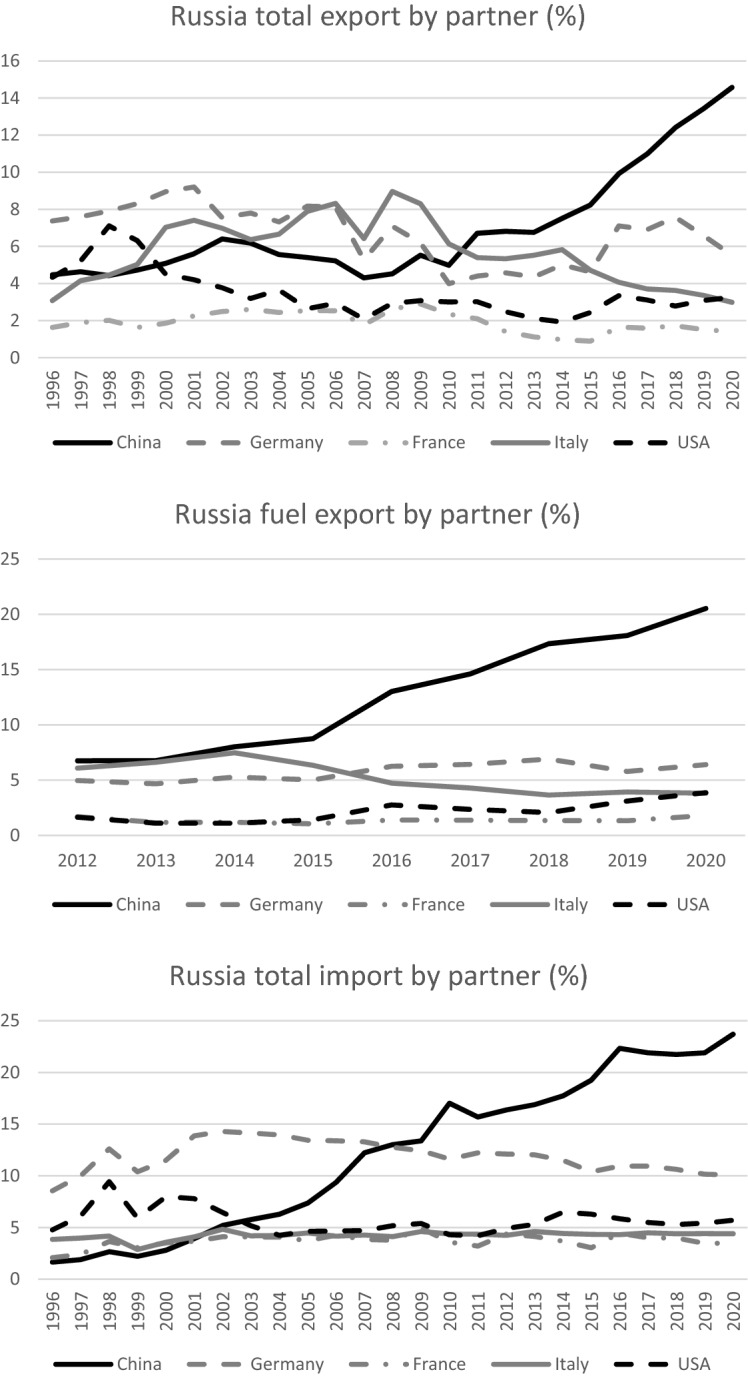
Russia trade by partners. Source: World Bank, WITS database

The European Union and Russia have close trade relations, but the respective positions are very asymmetric, both in terms of the relative importance and in terms of the type of flows exchanged. The EU considered as a whole is by far the main trading partner of Russia, even if its relevance is declining. In 2018, the EU received about 58% of total Russian exports, while in 2020, the EU accounted for 37.3% of the country’s total trade in goods with the world. 36.5% of Russia’s imports came from the EU and 37.9% of its exports went to the EU. Within the EU, Germany and Italy exchange the largest amounts with Russia. Germany has been the most important country in Russian trade until 2007, but it was overcome by China’s rapid trade growth. China, now the main trading partner of Russia in terms of individual countries, receives an increasing share of exports currently around 15%, and it is the origin of nearly one quarter of Russian imports (Fig. 7).

Considering Russia’s fuel exports only, Italy and Germany shifted the first and second position in the past decade, but China is now the main export market for Russia also for these commodities, receiving nearly 20% of Russian exported fuels.

From the point of view of the EU instead, Russia represents about 5% of its total trade, and it is its fifth partner. In 2021, the total trade in goods between the EU and Russia amounted to €257.5 billion. The EU’s imports were worth €158.5 billion and were dominated by fuel and mining products – especially mineral fuels (€98.9 billion, 62%), wood (€3.16 billion, 2.0%), iron and steel (€7.4 billion, 4.7%), fertilizers (1.78 bn, 1.1%). The EU’s exports to Russia in 2021 totaled €99 billion. They were led by machinery and equipment (€19.5 billion, 19.7%), motor vehicles (€8.95 billion, 9%), pharmaceuticals (€8.1 billion, 8.1%), electrical equipment and machinery (€7.57 billion, 7.6%), as well as plastics (€4.38 billion, 4.3%).

An asymmetry between EU and Russia is present not only considering gross trade flows, like in above-mentioned figures, but also considering trade in value added and the reciprocal contribution to the production processes. Overall, the value added of non-EU origin in EU final demand in 2018 was about 13%, based on OECD TiVA data. Specifically, the share of Russian value added in EU final demand in 2018 was about 1%, substantially constant over the past decade. In spite of the relevance of Russian energy materials and other specific raw materials in EU industrial production, the Russian value added in EU final demand is about half of the one originated in China, and one third of the value added originated in the USA. As mentioned, considering the Russian value added in EU gross exports, the share is only slightly higher, 1.3%. Instead, the European value added in Russia final demand was about 7%, and total foreign value added contributed by over 25% to Russian final demand, suggesting a relevant dependency of this economy on foreign trade.

While the EU has a trade deficit with Russia in terms of goods, it runs a large surplus considering services. Two-way trade in services between the EU and Russia in 2020 amounted to €29.4 billion, with EU imports of services from Russia representing €8.9 billion and exports of services to Russia accounting for €20.5 billion. In 2019, the EU was the largest investor in Russia. The EU foreign direct investment (FDI) outward stock in Russia in 2019 amounted to €311.4 billion, while Russia’s FDI stock in the EU was estimated at €136 billion.

### EU energy dependency

**Fig. 8:**
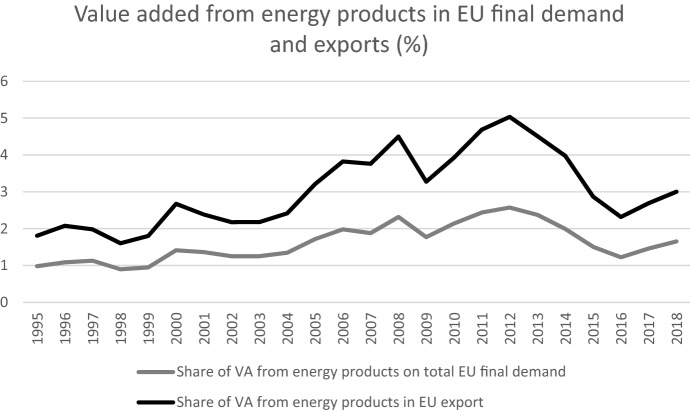
Energy intensity of EU final demand and EU gross exports. Source: OECD TiVA database

Like all modern economies, the EU production system and final demand require energy as an essential production input. The energy intensity of most economies, including the EU, has generally declined in the past decades thanks to technological progress, and to policies put in place to limit the environmental impact of human activities. Between 1990 and 2017, the EU's energy intensity i.e. the ratio between its gross inland energy consumption and its GDP decreased by 37%, according to the European Environment Agency, with a nearly continuous decline. More unstable is the trend of another indicator, the share of value added from energy products in EU final demand, which is affected also by price volatility: the share increases from the mid-1990s until 2012, and then declines (see Fig. 8). A similar pattern of the share of value added from fuels is observed also for EU gross exports.

The EU is not self-sufficient for its own energy consumption, and it needs energy that is imported from third countries. In 2020, the main imported energy product was petroleum products (including crude oil, which is the main component), accounting for almost two thirds of energy imports into the EU, followed by natural gas (27%) and solid fossil fuels (5%). The sources of these energy products for the EU are quite concentrated. In 2020, based on Eurostat data,^10^ almost three quarters of the extra-EU crude oil imports came from only seven countries, with Russia as the primary source at 29%, followed by USA (9%), Norway (8%), Saudi Arabia and the United Kingdom (both 7%), Kazakhstan and Nigeria (both 6%). Similarly, over three quarters of the EU's imports of natural gas came from only four countries, with a different geographic dispersion, but again with Russia as the main origin (43%), trailed by Norway (21%), Algeria (8%) and Qatar (5%). Also more than half of solid fossil fuel (mostly coal) imports originated from Russia (54%), followed at a distance by the United States (16%) and Australia (14%). It is important to underline that the oil market is a global one, and the geographic origin of crude oil is highly substitutable, while the gas market tends to be more regional. Market concentration is even higher from the Russian perspective: the EU27 and UK together account for over 63% of Russia’s fossil fuel exports, and with US, Turkey and Japan, the share of Russia exports increases to 80%.

**Fig. 9:**
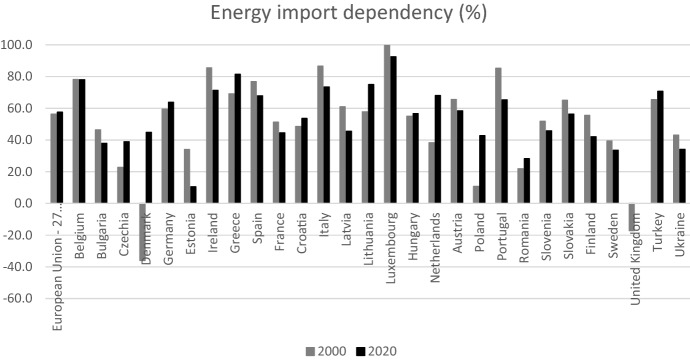
Energy import dependency: ratio of imported fuels on fuels consumption in selected countries. Source: Eurostat

The dependency rate is an indicator measured by the share of net imports (imports–exports) in gross inland energy consumption (meaning the sum of energy produced and net imports), showing the extent to which an economy relies upon imports in order to meet its energy needs. In 2020 for the EU, the dependency rate was equal to 58%, which means that more than half of the EU’s energy needs were met by net imports (Fig. 9). This rate is lower compared with 2019 (60%), which is partly linked to the COVID-19 economic crisis, however it is still slightly higher compared with 2000 (56%). Across the EU member states, the import dependency rate varies remarkably, and there is also variation in the dependency trend, with some countries (such as Italy) lowering their foreign dependency, and others increasing it (like Germany).^11^

Given this level of dependency, there are many potential economic effects of a reduction in supply and an increase in prices of fossil fuels that may occur as a consequence of the Russia-Ukraine conflict (for a recent overview, see Blanchard & Pisani-Ferri, 2022). Following the recent shock created by this war, estimates have been provided using Computable General Equilibrium (CGE) models to try to assess the impact of a cut of Russia’s fuel exports both on Russia and on the EU (see Chepeliev, 2020 and Chepeliev et al., 2022). The estimates suggest that the impact would be very strong on the Russian economy, but of course, it would also hurt the EU economy and other energy importers imposing import restrictions. Measuring the cost of switching energy sources and the relevance of the resulting impact is crucial to allow for the appropriate policy decisions. An important element considered in CGE models in evaluating the impact is the effect of such a policy is the environmental quality. The EU recently announced the so-called Green Deal, setting ambitious mitigation goals and establishing a target of reducing greenhouse gas emissions by 55% by 2030 relative to the 1990 level. To reach this goal, a major reduction in fossil fuel consumption will need to take place in the coming years, and a forced reduction of fuel imports from Russia might provide an additional incentive in this direction.

The ENVISAGE global computable general equilibrium model (van der Mensbrugghe, 2019) used by Chepeliev et al. (2022) moves from a baseline scenario of macroeconomic, energy, and emission profiles that is based on the continuation of current trends until 2030. The model then simulates a scenario whereby the EU and other high-income countries impose restrictions in the form of increasing tariff barriers on imports of fossil fuels from Russia starting from 2022. The predicted reduction in imports of fossil fuels by the EU ranges from 40 to 60% in 2022 and reaches 70–90% in 2024 (relative to the baseline). The interesting result of such simulation is that such restrictions could come at a modest long-run cost to the EU. The cumulative reduction in real income is less than 0.4% in 2030. This translates into a slowdown in the income growth rate of only 0.04% per year – instead of growing at 2.18% per year, the EU’s real income would be growing at 2.14% per year over the period 2022–2030.

Of course, the effects are not evenly distributed in time and across countries. The immediate impacts (during the first few months) of restrictions on Russia’s fossil fuel exports are likely to be non-trivial, and therefore more politically complex to deal with. EU households’ real income could drop by 0.3–0.6% (relative to the reference case), because of the rapid increase in energy prices, up to 6.8–8%. Energy price increases have very different impacts across EU countries because of the industry composition, the above mentioned dependency ratio and consumption habits.^12^ On average, according to Eurostat data, the poorest EU households spend 11.3% of their income on energy and transport fuels, but this share substantially varies across countries, being as low as 6% in Sweden and exceeding 23% in Slovakia. At the same time, the model finds substantial environmental co-benefits of such a move, thanks to reduced CO_2_ emissions, dropping by 3.1% in 2022 and reaching—5.5% in 2030 compared to the baseline.

As already highlighted by the strong correlation observed between fuel revenues and Russian GDP, the model predicts that EU cut of fuel imports would be a major burden on the Russian economy, slowing economic growth, reducing government budget revenues, and substantially decreasing the country’s ability to finance military operations. The immediate reduction in real income would be almost 6% relative to baseline (in 2022) and would reach 8% percent by 2030. The model estimates also suggest that by 2030 the cumulative loss in real income for Russia would exceed $1.1 trillion, while cumulative export revenue losses from reductions in fossil fuel exports would amount to almost $1.4 trillion.

The model is also used to explore a set of sensitivity scenarios, with limits to the trade substitution possibilities, likely to occur in the short run. To offset the supply problems that have emerged and the pressures that Russian energy issues have created, many European countries have been trying to diversify their energy sources away from Russia. Yet it is not easy for large economies to switch suppliers in the midst of a tight energy market. The US, Canada, Qatar, Norway, and Algeria have been asked to expand their energy supply to Europe, and new supply in liquified natural gas (LNG) has helped substitute somewhat for Russian gas. But Europe and the world are currently facing serious upstream and downstream constraints.

Under the more restrictive hypotheses of constraints similar to the ones observed, the model predicted reduction in imports of natural gas by the EU ranges between 79 and 90%, while for the case of crude oil the reduction is between 68 and 82%. Under such scenarios, real income in the EU decreases by 0.3–0.6% relative to the reference case, while energy prices for EU households on average increase by 6.7–8%. While these are certainly non-negligible effects, also in this case the impact on the EU economies is likely to be smaller than the one of the pandemic crisis. At the same time, such restrictions could result in substantial environmental co-benefits through reductions in CO_2_ and air pollutant emissions, with mitigation costs that are comparable to further increases in the EU Emissions Trading Scheme (ETS) carbon pricing.

## Conclusions

The data reported in the above analysis confirm the uneven growth and economic weakness of Russia, especially in comparison to other emerging economies. Such weakness is related to the reliance of Russia on its fuel exports that, in spite of its large size, allowed the country to develop a Dutch disease effect. Russia did not cultivate a competitive manufacturing sector and did not fully enter into the international trading system, even being a WTO member for 10 years now, and its participation in GVCs is very distorted.^13^

Such a high reliance on fuels exports has brought about a high dependence on foreign demand and economic fluctuations. Even if fuels are important in world trade and the energy dependency of large markets such as the European one is big, fuels are commodities with a large volatility and a high price elasticity in the medium-long run, and it is very difficult to build a stable development path based exclusively on this industry. According to World Bank data, at the world level the share of fuel imports on total merchandise imports is fluctuating, but it declined from an average around 16% in the period 2005–2014 to the average share of 11% in the past 5 years. A similar change can be observed also for the share of EU fuel imports. Overall, the evidence suggests that Russia’s economic dependency on the EU is higher than the reverse EU dependency on Russia. Even if the worst-case scenario of a complete cut of Russia’s fuel exports can generate a significant impact on the EU economy, this appears to be bearable, especially if additional adaptation and diversification efforts from EU member states are put in place. This would also require policies that reduce the economic burden on the most vulnerable sectors and citizens. Therefore, the consequences of such a shock could generate political concerns and strains among EU members even in presence of a manageable economic damage.

EU and many other advanced countries’ efforts to reduce their consumption of fossil fuels are increasingly undermining Russia’s position in world trade. Many oil exporters have been clearly aware of this risk for years now, and started already to diversify their economies. Unfortunately, the time of a full substitution of fossil fuels is still quite remote. Even if a substitution toward sustainable energy sources is underway, the International Energy Agency reported that investment in clean energy must triple to achieve a global energy transition. While public and private sector investments seem to have accelerated since the invasion of Ukraine with notable announcements in offshore wind, solar, and nuclear these investments will be slow and insufficient to meet the world’s energy security and transition goals in the short run. Also, a relevant problem for the energy transition is that the emerging clean energy era will not allow for the dispersion of energy producers and consumers that the world sees today, given the technical difficulties associated with transporting renewable energy. This means that all countries, particularly high-consuming rich countries that have long imported their energy needs, will have to accept the ‘deglobalization of global energy markets’ and increase their reliance on local and regional energy production. In order to return to globalized energy markets, new technologies and a new infrastructural landscape must emerge.

In the meanwhile, what will happen to Russia and its economic relations with the EU and with other countries? In a rapidly changing scenario for the world economy and with high risks of increased fragmentation of world markets also because of the growth of “special interest policies” (on this, see also Mariotti, 2022, in this same Special Section), this is a very relevant and difficult question. Because of its size, and especially its historical and military role (see also George & Sandler, 2022, in this same Special Section), Russia might still deeply affect world balances.

Accelerating an already observed trend, Russia might turn increasingly to China as a trade partner, less concerned with environmental issues and with different geopolitical priorities. Even if the two countries are dramatically different in many respects, trade models suggest that they share a number of important economic complementarities. Russia could sustain the Chinese growth through an abundant supply of fuels, agricultural commodities and raw materials, while China can provide labor force, technology and consumption goods and services to Russia, and they can both benefit from the reciprocal large markets. A cooperation between the two countries will certainly reinforce their economic dominance across Asia, the faster developing area of the world, and possibly strengthen their position in Africa. This conceivable evolution could cause relevant consequences in terms of world market fragmentation, deepening the division between the so-called advanced countries, especially in North America and Europe, and different areas of the world, and diminishing the role of many global international institutions. While the economic analysis might support this hazardous perspective, there are relevant political hurdles to this type of cooperation. Because of their strong nationalism and their historical rivalry, none of the two countries is likely to accept an alliance dominated by the other, and as shown many times by game theory, a perfectly balanced cooperation is almost impossible to reach and highly unstable. A cooperation between the two large countries is more likely to last in presence of a weakened Russia, sustained but somehow controlled by China. China might therefore indirectly benefit by the policies put forward by the EU and other countries to weaken Russia’s economy.

In the end, the short-term economic consequences of the conflict in Ukraine and the economic split between Russia and the EU are negative, but manageable for the EU and for the rest of the world. Possibly more serious are the medium-term economic consequences and impact on world trade, with shifting equilibria and increasing uncertainty and risks of fragmentation. Finally, one of the long-term costs might be the loss of Russia economic potential and resources for world markets.

## Footnotes

1 Hundreds of papers published especially in the 1990s studied the economic problems of transition economies. For a general overview of the main issues and a comparison between Russia and other transition economies, see for example Johnson et al., 1997; Schleifer, 1997; EBRD, 1999.

2 Deardorff’s Glossary of International Economics (2006) defines the so-called “Dutch disease” as the adverse effect on a country's other tradable industries when one industry's exports boom, causing real appreciation. Named for the effects of natural gas discoveries in the Netherlands, and most commonly applied to effects on manufacturing of exports of natural resources. (http://www-personal.umich.edu/~alandear/glossary/intro.html).

3 A transition economy is characterized by the process of converting from a centrally planned, non-market economy to a market economy. See Deardorff’ s Glossary of International Economics (2006).

4 Over the period 1991 to 2020, the correlation between GDP per capita in Russia and the level of oil prices is 0.78, while the correlation between GDP per capita growth and oil prices is 0.41 (see Fig. 1).

5 There is no formal definition of CEECs, but normally, the group of countries classified as belonging to Central and Eastern Europe includes Poland, Hungary Czech Republic, Slovakia, Romania, Bulgaria and the Baltic republics of Estonia, Latvia and Lithuania. Some of the countries belonging to the former Yugoslavia, such as Slovenia and Croatia can be included as well.

6 According to the World Population Review (www.worldpopulationreview.com), between 1993 and 2008, Russia saw a considerable decline in its population from 148.37 million to 143.25, because of low birth rates and abnormally high death rates. Since then, thanks also to the improving economic conditions, the population has increased to the current 144.71 million (but the growth rate has been negative, − 0.2%, in the last 2 years). The Russian population is expected to have reached its peak at the end of 2020 and is projected to start declining again, as Russia has one of the lowest fertility rates in the world and one of the oldest populations in the world. It is estimated that Russia will fall from being the 9th most populous country to 17th by 2050.

7 Other countries, like Vietnam or Jordan, experienced an increase in their export shares after joining the WTO. The economic literature has investigated the trade effects of WTO membership, with some discordant results. Overall, the empirical evidence supports a generally positive effect of the WTO membership on a country’s trade flows (Chang & Lee, 2011; Dutt, 2020; Dutt et al., 2013).

8 Recently, several studies such as Acemoglu et al. (2012) and Carvalho (2014) proposed theoretical models in which the influence of individual firms or sectors on aggregate economic outcomes is determined by their eigenvector centrality. Jackson and Nei (2015) use network analysis and network features to study the relationship between international trade and wars.

9 The PageRank indicator (originally developed to evaluate the centrality and relevance of web pages, see Page and Brin, 1998) belongs to the class of eigenvector centralities and it is now widely used. From the algorithm used for its computation, it is possible to see that the PageRank centrality for a given country i becomes higher if: the number of country i’s partners increases; country i's trade increase; PageRank for country i's partners increases.

10 See Eurostat https://ec.europa.eu/eurostat/cache/infographs/energy/bloc-2c.html.

11 For a more extensive discussion on the EU energy dependency, see Sturm (2022) in this same Special Section.

12 Different industries across the EU have quite different levels of energy intensity, and they are likely to be impacted very differently by a supply and price shock. As shown by Costa et al. (2022), it is quite complex to assess how the shocks will propagate throughout the economy, as not necessarily the most affected sectors are the ones generating the strongest impact, because this depends crucially on the relative position of each sector in the production system.

13 Certainly, there are other possible—also non-economic—reasons for Russia’s fragile economic performance, such as a super-power syndrome (Chebankova, 2017; Krickovic, 2014) fearing too much trade interdependence, many institutional weaknesses, a negative demographic evolution, but all these possible causes are not analyzed here in order to focus on trade and economic integration.

## References

[CR1] Aaronson S, Abouharb M (2014). Does the WTO help member states improve governance?. World Trade Review.

[CR2] Acemoglu A, Carvalho VM, Ozdaglar A, Tahbaz-Salehi A (2012). The network origins of aggregate fluctuations. Econometrica.

[CR3] Alexeev M, Weber S (2013). The Oxford handbook of the Russian economy.

[CR4] Antràs P, Chor D (2021). Global value chains. NBER Working Paper.

[CR5] Antràs P, Helpman E (2004). Global sourcing. Journal of Political Economy.

[CR6] Aturupane C, Djankov S, Hoekman B (1999). Horizontal and vertical intra-industry trade between Eastern Europe and the European Union. Weltwirtschaftliches Archiv.

[CR7] Balassa B (1965). Trade liberalization and 'revealed' comparative advantage. Manchester School.

[CR8] Baldone S, Sdogati F, Tajoli L (2001). Patterns and determinants of international fragmentation of production: Evidence from outward processing trade between the EU and Central Eastern European countries. Weltwirtschaftliches Archiv.

[CR9] Beck R, Kamps A, Mileva E (2007). Long-Term Growth Prospects for the Russian Economy. ECB Occasional Paper No..

[CR10] Behzadan N, Chisik R, Onder H, Battaile B (2017). Does inequality drive the Dutch disease? Theory and evidence. Journal of International Economics.

[CR11] Benedictow A, Fjærtoft D, Løfsnæs O (2013). Oil dependency of the Russian economy: An econometric analysis. Economic Modelling.

[CR12] Blanchard, O. J., & Pisani-Ferry, J. (2022), *Fiscal support and monetary vigilance: Economic policy implications of the Russia-Ukraine war for the European Union*, Peterson Institute for International Economics, Policy Brief 5–22.

[CR13] Borin, A., Conteduca, F. P., Di Stefano, E., Gunnella, V, Mancini, M., & Panon, L. (2022), *Quantitative assessment of the economic impact of the trade disruptions following the Russian invasion of Ukraine*, Occasional Paper Series, Banca d’Italia, forthcoming.

[CR14] Borin, A., & Mancini, M. (2019). *Measuring What Matters in Global Value Chains and Value-Added Trade*, Policy Research Working Paper No. 8804, World Bank, Washington DC.

[CR15] Borin, A., Mancini, M., & Taglioni, D. (2021). *Countries and Sectors in GVCs*, Policy Research Working Paper No. 9785. World Bank, Washington, DC.

[CR16] Brock GJ (1998). Foreign direct investment in Russia's regions 1993–95. Why so little and where has it gone?. Economics of Transition.

[CR17] Buck T, Filatotchev I, Nolan P, Wright M (2000). Different paths to economic reform in Russia and China: Causes and consequences. Journal of World Business.

[CR18] Buiter WH (2000). From predation to accumulation? The second transition decade in Russia. Economics of Transition.

[CR19] Carvalho VM (2014). From micro to macro via production networks. Journal of Economic Perspectives.

[CR20] Chang P-L, Lee M-J (2011). The WTO trade effect. Journal of International Economics.

[CR21] Chebankova E (2017). Russia’s idea of the multipolar world order: Origins and main dimensions. Post-Soviet Affairs.

[CR22] Chepeliev M (2020). GTAP-power data base: Version 10. Journal of Global Economic Analysis.

[CR23] Chepeliev, M., Hertel T., & van der Mensbrugghe, D. (2022). Cutting Russia’s fossil fuel exports: Short-term pain for long-term gain, *VoxEU*.

[CR24] Connolly, R., & Hanson, P. (2016). *Import substitution and economic sovereignty in Russia*, Research Paper, Chatham House, London.

[CR25] Copeland DC (2015). Economic Interdependence and War.

[CR26] Corden WM, Peter Neary J (1982). Booming sector and de-industrialization in a small open economy. Economic Journal.

[CR27] Costa S, Sallusti F, Vicarelli C (2022). Trade networks and shock transmission capacity: A new taxonomy of Italian industries. Journal of Industrial and Business Economics.

[CR28] Cristea AD, Miromanova A (2022). Firm-level trade effects of WTO accession: Evidence from Russia. Review of International Economics.

[CR29] De Benedictis L, Nenci S, Santoni G, Tajoli L, Vicarelli C (2014). Network analysis of world trade using the BACI-CEPII dataset. Global Economic Journal.

[CR30] De Benedictis L, Tajoli L (2008). Similarity in trade structures, integration and catching-up. Economics of Transition.

[CR31] De Benedictis L, Tajoli L (2018). Global and local centrality of emerging countries in the world trade network. Networks of international trade and investment: Understanding globalisation through the lens of network analysis.

[CR32] Deardorff AD (2006). Glossary of international economics.

[CR33] Domínguez-Jiménez, M., & Poitiers, N. (2020), *FDI another day: Russian reliance on European investment*, Policy Contribution 03/2020, Bruegel

[CR34] Dutt P (2020). The WTO is not passé. European Economic Review.

[CR35] Dutt P, Mihov I, Van Zandt T (2013). The effect of WTO on the extensive and the intensive margins of trade. Journal of International Economics.

[CR36] EBRD (1999). Transition report 1999.

[CR37] Ericson R (1991). The classical soviet-type economy: Nature of the system and implications for reform. Journal of Economic Perspectives.

[CR38] Ericson RE (1999). The structural barrier to transition hidden in input-output tables of centrally planned economies. Economic Systems.

[CR39] Erokhin V (2017). Self-sufficiency versus security: How trade protectionism challenges the sustainability of the food supply in Russia. Sustainability.

[CR40] George J, Sandler T (2022). NATO defense demand, free riding, and the Russo-Ukrainian war in 2022. Journal of Industrial and Business Economics.

[CR41] Gordon, N. (2022), *The EU Goes After Russian Oil Sales to Europe—With an Eye on a Larger Target*, Carnegie Endowment for International Peace.

[CR42] Gorodnichenko Y, Svejnar J, Terrell K, Katherine (2010). Globalization and innovation in emerging markets. American Economic Journal.

[CR43] Hoekman B, Djankov S (1997). Determinants of the export structure of countries in Central and Eastern Europe. World Bank Economic Review.

[CR44] Isachenko TM (2013). The trade policy of Russia: latest developments and main priorities. Rivista Di Studi Politici Internazionali.

[CR45] Jackson MO, Nei S (2015). Networks of military alliances, wars, and international trade. Proceedings of the National Academy of Sciences.

[CR46] Johnson S, Kaufmann D, Shleifer A (1997). The unofficial economy in transition. Brookings Papers on Economic Activity.

[CR47] Keynes, J. M. (1920). *The economic consequences of the peace*. Harcourt, Brace, and Howe.

[CR48] Koopman R, Wang Z, Wei S (2014). Tracing value-added and double counting in gross exports. American Economic Review.

[CR49] Krickovic A (2014). Imperial nostalgia or prudent geopolitics? Russia's efforts to reintegrate the post-soviet space in geopolitical perspective. Post-Soviet Affairs.

[CR50] Kuboniwa M (2012). Diagnosing the ‘Russian Disease’: Growth and structure of the Russian economy. Comparative Economic Studies.

[CR51] Iapadre PL, Tajoli L (2014). Emerging countries and trade regionalization. A network analysis. Journal of Policy Modeling.

[CR52] Mariotti S (2022). A warning from the Russian-Ukrainian war: Avoiding a future that rhymes with the past. Journal of Industrial and Business Economics.

[CR53] Martin P, Mayer T, Thoenig M (2008). Make trade not war?. The Review of Economic Studies.

[CR54] McMillan J, Woodruff C (2002). The central role of entrepreneurs in transition economies. Journal of Economic Perspectives.

[CR55] Neuwirth RJ, Svetlicinii A (2016). The current EU/US–Russia conflict over Ukraine and the WTO: A preliminary note on (trade) restrictive measures. Post-Soviet Affairs.

[CR56] Ofer G, Drebentsov V, Blejer MI, Škreb M (1999). Trade, trade policy, and foreign-exchange regimes under transition: russia and the Dutch disease. Balance of payments, exchange rates, and competitiveness in transition economies.

[CR57] Shepotylo O, Tarr DG (2013). Impact of WTO accession on the bound and applied tariff rates of Russia. Eastern European Economics.

[CR58] Page L, Brin S (1998). The anatomy of a large-scale hypertextual Web search engine. Computer Networks and ISDN Systems.

[CR59] Sachs, J. D., & Warner, A. (1995). *Natural resource abundance and economic growth*, NBER Working Paper 5398, NBER.

[CR60] Shleifer A (1997). Government in transition. European Economic Review.

[CR61] Sturm, C. (2022). Between a rock and a hard place: European policy and complexity in the wake of the Ukraine War. *Journal of Industrial and Business Economics*

[CR62] Svejnar J (2002). Transition economies: Performance and challenges. Journal of Economic Perspectives.

[CR63] Tajoli L, Felice G (2018). Global value chains participation and knowledge spillovers in developed and developing countries: An empirical investigation. European Journal of Development Research.

[CR64] van der Mensbrugghe D (2019). The environmental impact and sustainability applied general equilibrium (ENVISAGE) model. Version 10.01.

[CR65] Winkler, D., & Wuester, L. (2022). Implications of Russia’s invasion of Ukraine for its value chains, VoxEU.org

[CR66] Winkler D, Wuester L, Knight D, Ruta M (2022). The effects of Russia’s global value chain participation. The impact of the war in Ukraine on global trade and investment.

